# Simultaneous Determination of Fifteen Constituents of Jitai Tablet Using Ultra High-Performance Liquid Chromatography Coupled with Triple Quadrupole Electrospray Tandem Mass Spectrometry

**DOI:** 10.3390/molecules19021635

**Published:** 2014-01-28

**Authors:** Shuping Wang, Peng Fu, Lei Liu, Lingling Wang, Chengcheng Peng, Weidong Zhang, Runhui Liu

**Affiliations:** 1 School of Pharmacy, Second Military Medical University, Shanghai 200433, China; E-Mail: shupingwang2007@163.com; 2 Department of Pharmacy, Changhai Hospital, Second Military Medical University, Shanghai 200433, China; E-Mail: naizhe168@163.com; 3 College of Pharmacy, Nankai University, Tianjin 300071, China; E-Mail: mark_2004@163.com; 4 National Engineering Research Center for TCM, Shanghai 201203, China; E-Mail: pharmacy2001@163.com; 5 School of Pharmacy, Shanghai Jiao Tong University, Shanghai 200240, China; E-Mail: pypengchengcheng@126.com

**Keywords:** Jitai tablet, UHPLC-ESI-MS/MS, quality evaluation, TCMP

## Abstract

An ultra-high-performance liquid chromatography-electrospray ionization tandem mass spectrometry (UHPLC-ESI-MS/MS) method was developed for simultaneous determination of fifteen constituents in Jitai tablet (JTT), a complex Traditional Chinese Medicine prescription (TCMP) used in treating opiate addiction. Benefitting from a small particle size (1.8 µm) C18 column, accelerated analysis with satisfactory resolution, sensitivity and selectivity were achieved in a single run within 7 min with linear gradient elution of acetonitrile-0.1% (*v/v*) formic acid in water. The analytical signal was obtained by multiple reaction monitoring transitions via electrospray ionization source operating in both positive and negative ionization mode. The approach was validated for linearity, sensitivity, precision, repeatability, stability and recovery. All analytes showed good linearity over a wide concentration range (*r* > 0.99). The method limits ranged from 0.03 ng/mL to 19.35 ng/mL which are sensitive enough for quality control studies. The developed method was successfully applied to the simultaneous determination of fifteen constituents in JTT. In conclusion, our experimental results demonstrate that UHPLC-ESI-MS/MS is a useful approach for the overall quality assessment of complex TCMPs.

## 1. Introduction

Quality control in Traditional Chinese Medicine prescriptions (TCMPa) has made considerable progress with the development of analytical technology over the past decades [1,2,3]. Generally, publications mainly study simple TCMPs, while for complex TCMPs, which usually contain more than ten crude drugs, despite their remarkable pharmacological activities, relatively few reports on quality control can be found, presumably due to the great difficulty in quantifying the complex components present at micro or trace concentrations, varied physicochemical properties [4,5,6]. Additionally, the marker compound selection is another bottleneck for the quality control of complex TCMPs. It is well accepted that a TCMP is a complex system containing tens or even hundreds of different chemical constituents, so the proportion of each crude drug component is even smaller in a complex TCMP than in a regular TCMP. In such a situation, the marker compound selection for quality studies of a complex TCMP is usually affected by subjective assessments, empirical evidences or the commercial availabilities of reference standards. Many publications and official documents suggest their own different categories for seeking marker compounds, for instance, the eight categories suggested by Song *et al.* in their recently published paper [7], and the four categories established by the European Medicines Agency (EMA) [8]. However, all of these studies reference a single crude drug, and no methods could be found for TCMPs. Therefore, a practical solution for marker compound selection of complex TCMPs should not merely link data from quality control to clinical safety and efficacy, but also help providing evidence for the mechanism of action of this ancient medicinal substance.

Jitai tablet (JTT) was selected as a model complex TCMP in this study. JTT, composed of fifteen crude drugs including Rhizoma Corydalis, Radix Salviae Miltiorrhiae, Radix Angelicae sinensis, Rhizoma Chuanxiong, Semen Persicae, Flos Carthami, Radix Aconite, Radix Ginseng, Cortex Cinnamomi, Rhizoma Zingiberis, Semen Myristicae, Flos Daturae, Radix Aucklandiae, Lignum Aquilariae Resinatrm and Margarita, is approved for the treatment of opiate addiction by the State Food and Drug Administration of China. Clinical studies have indicated that this formula exhibits notable curative effects on opiate detoxification, including less harmful side effects, high safety and satisfactory effects in the inhibition of protracted withdrawal symptoms [9,10], and is effective in the rehabilitation of abnormal body functions induced by chronic drug use [11,12]. Previous studies have focused mainly on pharmacological studies, instead of quality control. Other than the Chinese Pharmacopeia, no report could be found on the quality control of JTT. In addition, the quantitative standard for JTT in the Chinese Pharmacopeia is only to quantify tetrahydropalmatine, which is definitely an unreasonable and unacceptable situation [13]. JTT consists of several types of components, and “multiple components hitting multiple targets and exerting synergistic therapeutic efficacies” is its unique mechanism of action. Our previous study [14] revealed that the constituents of JTT are numerous and diverse, including alkaloids, saponins, organic acids and flavonoids. Based on a large amount of pharmacological research, the tertiary and quaternary alkaloids from Rhizoma Corydalis are known to exhibit antiemetic, antiarrhythmic, antinociceptive, anxiolytic, vasodilator, and sedative but non-addictive tranquilizing effects [15,16,17,18]. The caffeic acid, ferulic acid, salvianolic acid A, salvianolic acid B, amygdalin and hydroxysafflor yellow A from the other included herbal medicines are also the important for treating opiate addiction owing to their antiemetic [19], antiinflammatory [20], antinociceptive [21], anxiolytic [22], and sedative tranquilizing effects [23]. Therefore, determination of these types of compounds is quite necessary and reasonable for the quality evaluation of the whole prescription.

Several analytical methods have been respectively employed for the quantitative and qualitative study of the various crude drugs in JTT, including HPLC, capillary chromatography (CE), high-speed counter-current chromatography (HSCCC), and gas chromatography (GC). HPLC with different spectral detectors (HPLC-UV, HPLC-DAD, HPLC-ELSD, *etc.*) are the most widely used methods owing to their simple and reliable characteristics [24,25,26,27,28]. However, these methods are time-consuming, costly and tedious, presumably due to the extreme complexity and diversity of the co-existing components. Moreover, the sensitivity and selectivity of these methods are insufficient for the accurate determination of compounds present in micro or trace concentrations. Ultra-high-performance liquid chromatography-electrospray ionization tandem mass spectrometry (UHPLC-ESI-MS/MS), which reduces the particle diameter in analytical columns from 5 µm to sub-2 µm, allows either greatly speeding up the analytical process by a factor of nine-fold while maintaining similar efficiencies or a theoretical nine-fold increase in efficiency for a similar run time, thus providing efficient separation and high selectivity by means of ultra-high pressure elution, high peak capacity and multiple ion detection based on selective ion fragmentation. In this study, a UHPLC-ESI-MS/MS method was developed and employed for the quality control study of JTT, and marker compound selection for the quality control of complex TCMPs was investigated as well.

## 2. Results and Discussion

### 2.1. Optimization of Sample Preparation

Variables such as the solvent, procedure, and time of extraction were optimized to achieve the best quantitative results. Different concentrations of methanol-water mixtures were studied, including 100% methanol, 75:25 methanol-water (*v/v*), 50:50 methanol-water (*v/v*), 25:75 methanol-water (*v/v*) and 100% water. 50:50 Methanol-water (*v/v*) showed the best extraction efficiency. Additionally, compared with refluxing and Soxhlet extraction, ultrasonic extraction was simple, reproducible, and effective. Moreover, the extraction time (15, 30, 60 min) was also evaluated. The results suggested that ultrasonication with 50:50 methanol-water (*v/v*) for 30 min was a simple and effective procedure for the extraction of the fifteen compounds As follow-up work, the fifteen analytes selected in this paper were not merely selected based on the results of the plasma pharmacochemistry study and bioactivity screening of JTT, but the compounds with suitable therapeutic, bioactive, characteristic, main, synergistic, correlative, toxic and analytical properties in treating opiate addiction. Their pharmacological activity was stated in the introduction section, and the criteria for analyzing for these fifteen compounds was stated in part “2.5 Marker Compound Selection for Quality Control of Complex TCMP”.

### 2.2. Optimization of UHPLC-ESI-MS/MS Conditions

Liquid chromatographic conditions such as stationary phase, mobile phase, column temperature and flow rate that could greatly affect the separation were investigated. A Zorbax Eclipse Plus C18 column (3.0 mm × 100 mm I.D, 1.8 µm, Agilent, Santa Clara, CA, USA) was chosen in the present study for its increased column efficiency and improved peak shape at high column temperatures. Different mobile phases (methanol-water, acetonitrile-water, methanol-acid aqueous solution and acetonitrile-acid aqueous solution) were examined and aqueous acetonitrile-acid solution exhibited the best separation. Indeed, the addition of 0.1% (*v/v*) formic acid in water of the mobile phase not only dramatically enhanced the abundance of [M+H]^+^ ions, but also eliminate the peak tailing of the target compounds in negative ion mode. Excellent separation was achieved when the column temperature was kept at 35 °C at a flow rate of 0.3 mL/min by gradient elution in a total analysis time of 7 min.

**Figure 1:**
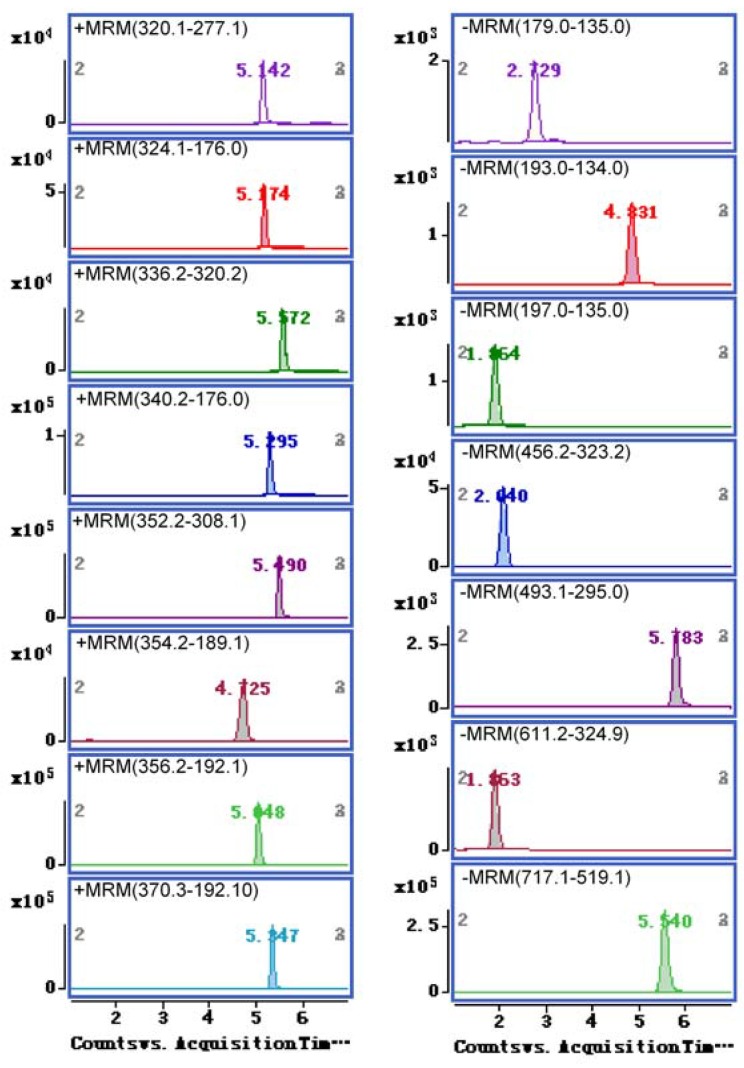
Representative MRM chromatograms of the fifteen compounds from JTT.

**Figure 2:**
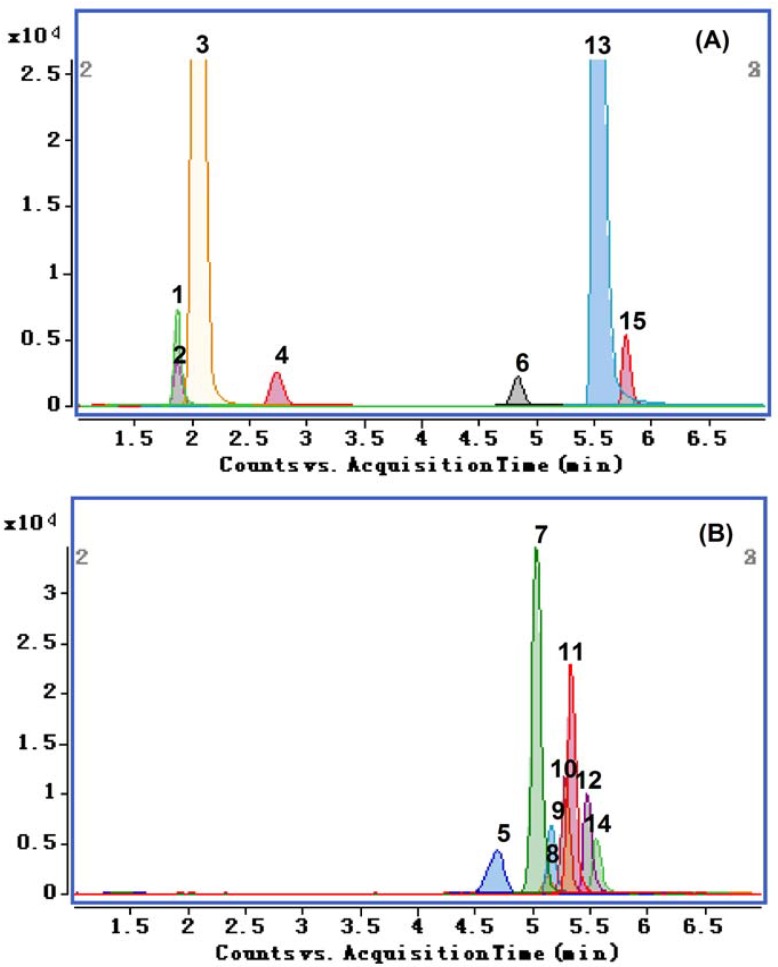
Total ion chromatograms of the fifteen compounds from JTT extract: (**A**) negative ion mode and (**B**) positive ion mode. 1. Hydroxysafflor yellow A; 2. Danshensu; 3. Amygdalin; 4. Caffeic acid; 5. Protopine; 6. Ferulic acid; 7. Tetrahydropalmatine; 8. Coptisine; 9. Tetrahydrocoptisine; 10. Tetrahydroberineper; 11. Corydaline; 12. Palmatine; 13. Salvianolic acid B; 14. Berberine; and 15. Salvianolic acid A.

To maximize the abundance of the molecular ions of the compounds, acquisition parameters (source temperature and ions spray voltage) were investigated, and the highest ion intensity for molecular ion of the analytes was achieved when the source temperature, the nebulizing gas (N_2_) pressure, the drying gas flow were set at 350 °C, 40 psi and 10 L/min, respectively. The MS/MS analysis was operated in both positive and negative ion mode. The representative MRM chromatograms and total ion chromatograms (TIC) of the fifteen compounds from JTT are shown in Figure 1 and Figure 2, respectively.

The following orders were employed to set up MRM transitions for all the target compounds: initially, the precursor and product ions of the compound were ascertained by infusing 1 µg/mL standard solutions in scan and product ions mode, respectively. On the basis of that, to get the richest relative abundance of precursor and product ions, the fragmentor energy and collision energy parameters were further optimized. Table 1 shows the optimized MS/MS transitions and energy parameters for all target compounds.

### 2.3. Method Validation

#### 2.3.1. Linearity, Limit of Detection (LOD) and Limit of Quantification (LOQ)

The linearity of the method was examined by analysis of a series of concentrations of standard solutions prepared in methanol containing at least eight non-zero concentrations. Each calibration curve was analyzed individually by fitting the area ratio response for each analyte, using least square weighted (1/x^2^) linear regression and excluding the point of origin. Nice linear relationships and good coefficients of determination (r > 0.99) were obtained over a 500-fold wide range for the fifteen compounds. The LOD was determined by successive analysis of methanol with decreasing amounts of standard solutions until a signal-to-noise ratio (S/N) 3:1 was reached, and the LOQ was adopted as the concentration of a compound giving S/N 10:1. Detailed information regarding calibration curves, linear ranges, LOD and LOQ is presented in Table 2. The value of LOD and LOQ could meet the requirements of simultaneous determination of the fifteen compounds.

#### 2.3.2. Precision, Repeatability and Stability

The intra-day precision for each compound was assessed by measuring a standard mixture solution composed of fifteen compounds six times a day, while inter-day precisions were evaluated twice a day on three consecutive days. The intra-day or inter-day precision was calculated as relative standard deviation (RSD) using one-way ANOVA with day as the grouping variable. The RSD ranges of the fifteen compounds were 0.30%–3.25% for intra-day precision and 0.65%–3.61% for inter-day precision.

In order to confirm the repeatability, five different working solutions prepared from the same sample (Batch no. 070101) were analyzed. Good repeatability with RSD less than 3.27% (*n* = 5) for the fifteen compounds is shown in Table 3.

Stability was tested at room temperature, the samples were analyzed in triplicate every 6 h within 24 h. Stability was expressed as the percentage decrease of sample solution: (Content in sample solution at 0 h-Content in sample solution at 24 h)/Content in sample solution at 0 h × 100. As shown in Table 3, RSD value was lower than 4.48% for the fifteen compounds.

#### 2.3.3. Recovery

In the recovery test, known amounts of mixed standard solution with three different concentration levels (high, middle and low) were added to known amounts of JTT samples (National Engineering Research Center for TCM Shanghai TCM Technology Co., Ltd. Shanghai, China; batch no. 070101). Then the mixed samples were extracted and analyzed with the established method, triplicate experiments were performed at each level. Recoveries were calculated by the formula: Recovery (%) = (Detected amoun − Original amount) / Spiked amount × 100.

As shown in Table 4, recoveries of the fifteen compounds ranged from 94.11% to 103.82% with RSD from 0.13% to 2.83%.

**Table 1:** Chromatogtaphic retention time, MRM parameters, fragmentor and collision energy for the fifteen compounds.

Analyte	Molecular Weight (Da)	Ionization Mode	Retention Time (min)	Q1 Mass (*m/z*)	Q3 Mass (*m/z*)	Dwell Time (ms)	Fragmentor (V)	Collision Energy (V)
Hydroxysafflor yellow A	612.2	ESI^−^	1.82	611.2	324.9	100	190	30
Danshensu	198.1	ESI^−^	1.86	197.0	135.0	100	90	4
Amygdalin	457.2	ESI^−^	2.04	456.2	323.2	100	160	6
Caffeic acid	180.0	ESI^−^	2.73	179.0	135.0	100	90	8
Protopine	353.1	ESI^+^	4.73	354.2	189.1	100	130	30
Ferulic acid	194.1	ESI^−^	4.83	193.0	134.0	100	80	10
Tetrahydropalmatine	355.2	ESI^+^	5.05	356.2	192.1	100	140	28
Coptisine	320.1	ESI^+^	5.14	320.1	277.1	100	140	38
Tetrahydrocoptisine	323.1	ESI^+^	5.17	324.1	176.0	100	150	30
Tetrahydroberineper	339.2	ESI^+^	5.30	340.2	176.0	100	130	25
Corydaline	369.2	ESI^+^	5.35	370.2	192.1	100	140	28
Palmatine	352.2	ESI^+^	5.49	352.2	308.1	100	130	28
Salvianolic acid B	718.2	ESI^−^	5.54	717.1	519.1	100	150	13
Berberine	336.1	ESI^+^	5.57	336.2	320.2	100	130	30
Salvianolic acid A	494.1	ESI^−^	5.78	493.1	295.0	100	130	10

**Table 2:** Regression data, LODs and LOQs for the fifteen compounds (*n* = 5).

Analyte	LOD (ng/mL)	LOQ (ng/mL)	Linear Range (ng/mL	Slope (Mean ± SD)	Intercept (Mean ± SD)	*r*
Hydroxysafflor yellow A	0.18	0.60	20.00–10,000.00	10.2902 ± 0.2564	2250.3307 ± 52.4467	0.9956
Danshensu	0.60	2.00	20.00–10,000.00	8.6778 ± 0.1746	−456.6715 ± 9.8327	0.9992
Amygdalin	1.34	4.46	200.00–100,000.00	16.1377 ± 0.3781	4149.1033 ± 82.7875	0.9978
Caffeic acid	0.32	1.07	1.50–750.00	54.9048 ± 2.0422	385.0904 ± 8.8482	0.9991
Protopine	0.08	0.27	3.00–1500.00	1908.4269 ± 30.4657	975.8435 ± 17.3396	0.9990
Ferulic acid	2.21	7.36	15.00–7500.00	2.2024 ± 0.0462	1946.4735 ± 14.9437	0.9920
Tetrahydropalmatine	0.04	0.13	3.00–1500.00	6601.3733 ± 95.4892	2954.9081 ± 56.2518	0.9993
Coptisine	0.05	0.17	3.00–1500.00	491.4143 ± 9.3921	236.4471 ± 4.6437	0.9993
Tetrahydrocoptisine	0.06	0.20	1.50–750.00	2399.6703 ± 42.722	955.3523 ± 15.3732	0.9996
Tetrahydroberineper	0.02	0.07	1.50–750.00	8906.0226 ± 101.7735	1590.6877 ± 43.2583	0.9994
Corydaline	0.09	0.30	3.00–1500.00	4017.0902 ± 99.8759	537.0929 ± 10.2247	0.9994
Palmatine	0.02	0.07	3.00–1500.00	2643.7965 ± 55.2618	−495.5594 ± 10.7284	0.9997
Salvianolic acid B	5.81	19.35	500.00–250000.00	30.7107 ± 1.2739	606660.6878 ± 1298.4689	0.9956
Berberine	0.01	0.03	1.50–750.00	3759.8114 ± 98.3726	2643.4459 ± 67.7739	0.9991
Salvianolic acid A	3.45	11.49	15.00–7500	7.4196 ± 0.5444	2354.9524 ± 78.1819	0.9916

**Table 3:** Precision, repeatability and stability for the fifteen compounds.

Analyte	Intra-day (RSD *n* = 6)	Inter-day (RSD *n* = 6)	Repeatability (RSD *n* = 6)	Stability (24 h) (RSD *n* = 6)
Hydroxysafflor yellow A	2.03	3.60	2.90	3.28
Danshensu	0.71	3.01	3.27	2.09
Amygdalin	1.44	2.95	2.05	2.01
Caffeic acid	1.23	2.40	3.05	1.33
Protopine	0.49	0.68	1.45	2.19
Ferulic acid	1.44	3.08	3.09	4.48
Tetrahydropalmatine	0.61	0.95	1.85	3.82
Coptisine	0.74	0.94	1.79	3.75
Tetrahydrocoptisine	0.36	0.65	2.09	3.41
Tetrahydroberineper	0.41	1.24	1.86	2.99
Corydaline	0.30	0.95	1.89	2.75
Palmatine	1.00	0.84	1.57	2.37
Salvianolic acid B	1.82	3.61	3.23	4.47
Berberine	0.31	1.00	0.81	2.49
Salvianolic acid A	3.25	2.00	3.10	3.22

**Table 4:** Recovery of the fifteen compounds at three levels.

Analyte	Original (ng)	Spiked (ng)	Detection (ng)	Recovery (%)	RSD (%)
Hydroxysafflor yellow A	99.55	80	179.19	99.55	1.80
	99.55	100	200.10	100.55	0.19
	99.55	120	219.01	99.55	0.64
Danshensu	36,713.81	29,600	66,084.86	99.23	1.52
	36,713.81	37,000	74,372.45	101.78	0.65
	36,713.81	44,400	80,770.39	99.23	1.19
Amygdalin	606.20	480	1098.17	102.49	2.83
	606.20	600	1192.46	97.71	1.42
	606.20	720	1342.21	102.22	1.84
Caffeic acid	1018.24	800	1823.74	100.69	0.55
	1018.24	1000	2025.88	100.76	1.80
	1018.24	1200	2251.24	102.75	1.78
Protopine	173.99	140	311.99	98.57	0.51
	173.99	180	346.86	96.04	0.79
	173.99	225	388.99	95.56	1.55
Ferulic acid	10,800.76	8640	19,441.37	100.01	0.66
	10,800.76	10,800	21,686.07	100.79	0.89
	10,800.76	12,960	23,761.68	100.01	0.58
Tetrahydropalmatine	223.40	180	402.12	99.29	0.51
	223.40	225	446.69	99.24	0.95
	223.40	270	489.48	98.55	0.79
Coptisine	222.30	180	398.15	97.69	1.42
	222.30	225	449.66	101.05	0.50
	222.30	270	497.07	101.77	0.25
Tetrahydrocoptisine	96.60	80	175.89	99.11	0.13
	96.60	100	198.22	101.62	0.71
	96.60	120	209.53	94.11	0.71
Tetrahydroberineper	47.47	40	88.44	102.43	0.21
	47.47	50	99.38	103.82	0.22
	47.47	60	108.43	101.60	0.80
Corydaline	154.16	120	277.49	102.78	0.26
	154.16	150	296.95	95.19	0.28
	154.16	180	339.16	102.78	0.23
Palmatine	223.44	180	402.19	99.31	1.51
	223.44	225	440.03	96.26	1.16
	223.44	270	491.56	99.30	0.44
Salvianolic acid B	1495.32	1200	2691.58	99.69	0.77
	1495.32	1500	2979.81	98.97	2.25
	1495.32	1800	3289.71	99.69	0.49
Berberine	52.89	40	93.08	100.48	0.55
	52.89	50	101.78	97.78	1.01
	52.89	60	113.77	101.47	1.01
Salvianolic acid A	848.31	680	1526.95	99.80	1.64
	848.31	850	1715.10	101.98	0.73
	848.31	1020	1866.28	99.80	0.62

**Table 5:** Content of the fifteen compounds in fifteen batches of JTT sample (µg/g).

Compounds	Batch No.													
	50,302	50,303	50,401	50,601	50,602	70,101	70,102	70,501	70,502	90,401	91,201	100,101	100,102	110,701
Hydroxysafflor yellow A	408.13	133.54	73.22	88.64	87.7	274.94	282.12	258.85	111.33	299.29	301.11	259.11	477.09	68.87
Danshensu	570.46	726.94	763.21	672.6	626.5	478.07	734.94	461.14	557.16	581.95	417.27	943.38	467.38	1406.02
Amygdalin	3566.33	3907.22	3928.31	4611.34	4533.97	4037.1	4618.81	5405.11	3782.52	7642.94	5825.54	5504.12	8527.1	4081.63
Caffeic acid	36.06	18.27	22.18	23.33	14.01	29.39	34.6	45.17	32.1	28.2	30.06	24.61	38.52	22.79
Protopine	135.37	87.81	90.38	87.33	85.6	108.84	94.09	80.63	81.92	36.42	69.17	68.25	35.8	66.78
Ferulic acid	155.6	82.59	98.93	103.46	85.93	157.09	172.73	211.41	151.8	230.84	273.67	259.4	330.46	192.62
Tetrahydropalmatine	177.55	152.42	164.02	112.17	119.27	160.45	109.38	119.25	102.69	43.33	81.81	64.45	38.96	81.2
Coptisine	253.52	141.21	149.96	132.52	110.31	184.39	138.94	121.61	123.71	36.43	75.06	64.15	33.94	52.13
Tetrahydrocoptisine	81.34	55.83	59.79	36.72	33.32	64.62	35.88	39.81	32.62	19.06	38.56	32.3	16.49	39.95
Tetrahydroberineper	22.81	19	23.05	12.58	18.69	26.86	12.25	13.1	11.08	5.64	11.99	10.18	4.3	11.35
Corydaline	132.19	98.41	122.47	51.16	67.91	124.4	45.23	61.93	40.66	17.06	57.79	50.51	17.15	51.77
Palmatine	193.22	104.11	98.49	131.12	93.58	146.23	163.81	136.34	135.15	45.25	79.9	80.54	46.17	75.24
Salvianolic acid B	20,674.32	2583.65	2655.05	15,835.48	2532.11	2135.44	14,172.63	9504.99	10,043.33	13,626.23	2166.91	5100.94	16,852.62	5460.88
Berberine	41.06	23.61	25.05	27.7	24.24	36.55	34.16	28.26	29.34	10.89	40.74	45.81	14.56	17.07
Salvianolic acid A	814.45	640.97	735.76	1017.09	998.76	666.93	1113.39	831.28	1112.67	717.89	329.76	196.28	509.48	385.02
Total amount	27,262.41	8775.58	9009.87	22,943.24	9431.9	8631.3	21,762.96	17,318.88	16,348.08	23,341.42	9799.34	12,704.03	27,410.02	12,013.32

### 2.4. Quantitative Analysis of Fifteen Compounds in JTT Samples

The content of the fifteen compounds in JTT was determined in triplicate by the established method. The results of the quantitative analyses are presented in Table 5. Amygdalin and salvianolic acid B were determined to be the top two compounds, with concentration ranges of 3,566.33–8,527.10 µg/g and 2,135.44-20,674.32 µg/g. Among the other analytes, salvianolic acid B, amygdalin, salvianolic acid A, danshensu, hydroxysafflor yellow A, ferulic acid, and palmatine were determined as the main active compounds in JTT. Comparatively, some trace but effective components such as corydaline, tetrahydrocoptisine, tetrahydroberineper and berberine were observed and simultaneously determined. The difference in the content of the compounds between different batches may be attributed to the different plant origins, sources, cultivated year, harvest time, geographical climate and environment.

### 2.5. Marker Compound Selection for Quality Control of Complex TCMP

Marker compound selection has always been the bottleneck in quality control of complex TCMPs due to the diversity of the components in the TCMPs and the complexity of their mechanism of action. Ideally, the rational marker compound selection for quality control of TCMPs should be strongly correlated to their safety and efficacy. As suggested by the EMA and related references, some categories of constituents are defined as marker compounds including principle (constituents that have known clinical activities), active (constituents that have some known pharmacological activities), negative (constituents that may have allergenic or toxic properties) and analytical (constituents that are chosen as markers for identification and quantitative determination) markers. However, the conventional method for discovery of marker compounds such as systematic chemical separation followed by pharmacological activity assay or bioassay guided chemical separation, is not only proven to be time-consuming, labor intensive, error-prone or costly, but also cannot explain the synergistic action of multiple components of TCMP. Song, *et al.* reported that the combination of RPCA with UHPLC-UV-QTOF MS was a reliable means to identify chemical markers for evaluating quality of herbal medicines. Nevertheless serious bioactivity trials should be performed to validate the suitability and feasibility of this developed method *in vitro*. In our previous study, the potential bioactive components of JTT were screened by a simplified methodology using an LC-DAD coupled with electrospray tandem mass spectrometry and plasma pharmacochemistry-based approach. As follow-up work, the fifteen analytes selected in this paper were not merely selected based on the results of the plasma pharmacochemistry study and bioactivity screening of JTT, but the compounds with suitable therapeutic, bioactive, characteristic, main, synergistic, correlative, toxic and analytical properties.

## 3. Experimental

### 3.1. Chemicals and Reagents

**Figure 3:**
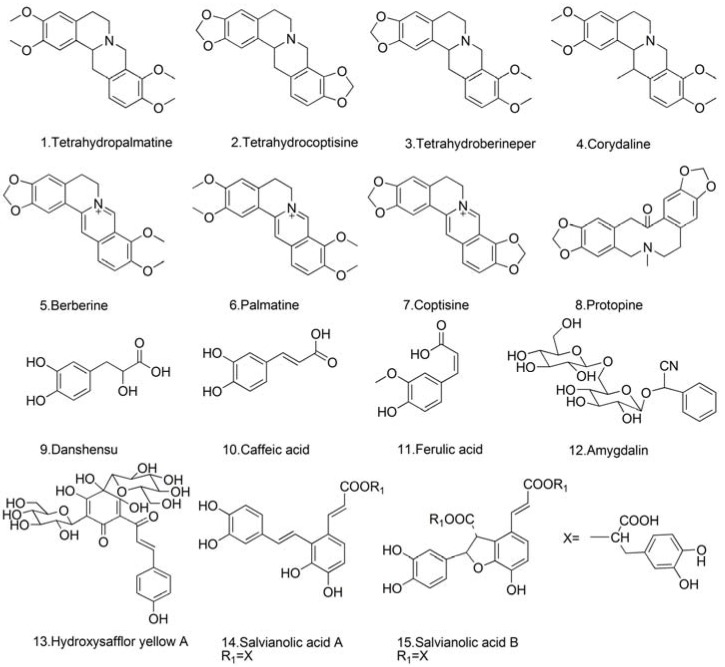
Chemical structures of the fifteen reference standards.

Reference standards of protopine, tetrahydropalmatine, coptisine, amygdalin, caffeic acid, danshensu, hydroxysafflor yellow A, ferulic acid were purchased from the National Institute for the Control of Pharmaceutical and Biological Products (Beijing, China). Tetrahydrocoptisine, tetrahydroberineper, corydaline, palmatine, berberine, salvianolic acid A, salvianolic acid B were obtained from Shanghai Sunny Biotech Co., Ltd (Shanghai, China). Their chemical structures are shown in Figure 3. Acetonitrile and methanol of HPLC grade were obtained from Merck (Darmstadt, Germany). Formic acid was purchased from Sigma-Aldrich (St. Louis, MO, USA). All aqueous solutions were prepared with ultra pure water produced from a Milli-Q50 SP Reagent Water System (Bedford, MA, USA). Other reagents were of analytical grade. JTT samples (Batch no.: 050301, 050302, 050303, 0504013, 0506013, 0506023, 0701013, 0701023, 0705013, 0705023, 0904013, 0912013, 1001013, 1001023, 110701) were kindly provided by the National Engineering Research Center for TCM Shanghai TCM Technology Co., Ltd. (Shanghai, China).

### 3.2. Sample Preparation

#### 3.2.1. Preparation of the JTT Analytical Sample

JTT samples were ground into a fine powder and sieved through a No. 40 mesh sieve to get a homogeneous size. Eighty mg was accurately weighed into a 10 mL volumetric flask. Methanol-water (50:50, *v/v*) was added and ultrasonically extracted for 30 min, and then cooled at room temperature. The same solvent was added to compensate for the lost volume to get a uniform suspension, then centrifuged at 13,500 g for 10 min (Universal 320R, Hettich, Germany) and the supernatant was filtered through a syringe filter (0.22 µm), and an aliquot (5 μL) of supernatant was subjected to UHPLC-ESI-MS/MS analysis.

#### 3.2.2. Preparation of Standard Solutions

The stock solution of the reference standards was prepared by dissolving requisite amount of each sample in methanol at a high concentration (200 µg/mL) except for salvianolic acid B and amygdalin at a concentration of (1,000 µg/mL). Different volumes of each stock solution were transferred into volumetric flasks and then diluted to volume to make working standard solutions with methanol. Calibration work solutions were prepared by diluting stock solutions with methanol, a series of final concentration were obtained. Calibration work solutions were stored at −20 °C until analysis.

### 3.3. Liquid Chromatography Conditions

Liquid chromatography was performed on an Agilent 1200 Series liquid chromatography (Agilent Technologies, Palo Alto, CA, USA), equipped with a quaternary pump with online degasser, autosampler and column oven. Chromatographic separation was performed on an Agilent Zorbax Eclipse Plus C18 column (3.0 × 100 mm I.D, 1.8 µm, Agilent). Analytical column was maintained at 35 °C and eluted with a mobile phase consisting of acetonitrile (A) and water containing 0.1% formic acid (B) using the following gradient program: 20% A→45% A at 0–1.0 min; 45% A at 1.0–2.5 min; 45% A→20% A at 2.5–4.0 min; 20% A at 4.0–7.0 min at a flow rate of 0.3 mL/min. The total run time was 7 min and the equilibrated time was 2 min. The effluent from the analytical column was directed from the waste to the mass spectrometer source after the first 1.0 min of each run.

### 3.4. Mass Spectrometric Conditions

Mass spectrometry was performed on an Agilent 6410B triple quadrupole mass spectrometer (Agilent Technologies), equipped with electrospray ionization (ESI) source and operating in both positive and negative ion mode. The drying gas temperature was maintained at 350 °C at a flow rate of 10 L/min, and the nebulizing gas (N_2_) pressure was set at 40 psi. The capillary was 3,500 V for negative mode and 4,000 V for positive mode. The dwell time was 100 ms, and mass analyzers Q1 and Q3 operated at unit mass resolution were used for all the multiple reaction monitoring (MRM) transitions. Compound-dependent parameters were listed in Table 1. Peak area obtained from MRM mode of the analytes was utilized for the construction of calibration curve, using weighted (1/x^2^) linear least squares regression of the concentrations and measured peak area. All data acquisition and analyses was processed with Agilent MassHunter Workstation Software (version B.03.01).

## 4. Conclusions

A sensitive, accurate and reliable UHPLC-ESI-MS/MS method has been developed and validated for the simultaneous determination of fifteen constituents from JTT. The analysis was carried out in a single 7 min run by an easy-to-use and high-throughput method. Compared with previous publications, the analytical results demonstrated better or comparable performance in terms of linearity, specificity, detection and quantification limits, precision and accuracy. In summary, UHPLC-ESI-MS/MS is a promising means for quality control and exploration of potential discrimination markers of complex TCMPs.
